# 

**Published:** 1890-12-13

**Authors:** 


### NOTICE TO CORRESPONDENTS.

All MS., Letters, Books for Review, and other matters intended for the Editor should be addressed The Editor, The Lodge, Porchester Square, London, W.

The Editor cannot undertake to return rejected M.S., even when accompanied by stamped directed envelope.

**Notice to Advertisers and Subscribers.**

All Advertisements, Orders for Copies of The Hospital, and Business Communications should be addressed to The Publisher, *not to the Editor*, The Hospital, 140, Strand, W.C.

**Bound Volumes of "The Hospital."**

Vol. VI., containing full page portraits of T.R.H. the Prince and Princess of Wales, and Miss Florence Nightingale, is still obtainable.

Vol. VII. and all the earlier volumes are also for sale.

Orders will be received for Vol. VIII., 4s. post free. Cases for binding may be had of the Publisher, at 1s. 6d. each.

**To Residents, Secretaries, and Matrons.**

The Editor will be glad to have the Regulations and each Report as issued by Asylums, Hospitals, Convalescent and Nursing Institutions, as well as those of other Charities, and an early copy in duplicate of all Appeals and Festival Notices, etc., addressed to *The Editor, The Lodge, Porchester Square, London, W.* Any superintendent, secretary, matron, or other chief official who regularly sends such information may be registered, on application to the Publisher, to receive free of cost a cover for binding The Hospital in half-yearly volumes.

### BURDETT'S HOSPITAL ANNUAL FOR 1890

May be obtained of the Publisher, The Hospital Offices, 140, Strand, W.C., price 2s. 6d. limp boards, or 3s. 6d. cloth. All orders must be accompanied by a remittance.

### BOOKS RECEIVED.

PEWTRESS AND CO.

"The Natural Food of Man." E. Densmore, M.D. (Cloth, 2s.: paper boards, 1s.)

SHAW AND SONS.
"The Law Relating to Medical Practitioners." Joseph Craven and Theodore Coppock. (7s. 6d.)

ARROWSMITH, F. W. (Bristol).

"The Demoniac" (Arrowsmith's Christmas Annual, 1890). Walter Besant.

KEGAN PAUL, TRENCH, TRUBNER, and Co.

"My Friends at Saût Ampelio." J. A. Goodchild.

MARLBOROUGH AND CO.
"Is Life Worth Living?" (Sixth Edition). John Clifford, M.A., D.D.

SCOTT, WALTER.
"The Criminal." (The Contemporary Science Series). Havelock Ellis.

### SCRAPS AND GLEANINGS.

ULVERSTON kept Hospital Saturday on December 6th.

A NEW hospital for Owens College has been offered by the Whitworth Trustees.

THE Walker Hospital issues a neat little report full of satisfactory information.

THE Adelaide Hospital, Dublin, is trying hard to clear off its debt and start fair in 1891.

THE Chaplain of the London Hospital appeals for funds for an organ for the new chapel.

DR. KOCH is said to have discovered cures for diphtheria and syphilis, which he will make public shortly.

THE caretakers of the Birkenhead Fever Hospital have sent in their resignations, which have been accepted.

HALIFAX INFIRMARY GOVERNORS have welcomed a scheme for a proposed provident dispensary in the town.

THE Rev. F. D. Perrott offers £10 towards establishing a Friendly Societies Convalescent Home for Suffolk.

MESSRS. WORTHINGTON AND ELGOOD, of Manchester, have been appointed architects to the new Halifax Hospital.

THE Earl of Ranfurly opened a s[illegible]e of work in aid of the Ulster Hospital for Women and Children on December 3rd.

SOME of the Governors of the Bury and West Suffolk Hospital are protesting loudly against the acceptance of the resignation of Dr. Image.

THE committee of the proposed Lan[illegible]hire Deaf and Dumb Institute have secured the £2,500 required t[illegible] claim the £5,000 grant of Miss Cross.

THE Rev. E. Carr Glynn lately presided over a drawing-room meeting held to consider the question of leprosy in the British Colonies, and the best means of alleviation.

DURING the month of November, smallpox attacked 2,100 persons, and caused 750 deaths in Madrid. The epidemic shows an increase during the first days of December.

AT the Chester Charities Inquiry it has been stated that the city companies, with one exception, existed solely for the purpose of deriving the benefit of the Owen Jones Charity.

CORK STREET FEVER HOSPITAL, Dublin, reports an increase in typhus cases. It will be strange if typhus, which we believe almost stamped out, regains a hold in the United Kingdom.

THE Rev W. T. McCormick states that he has sent three large boxes of goods to the lepers on Robben Island, and that he is very grateful for the numerous answers to his late appeal.

MR. WAINWRIGHT, the new treasurer at St. Thomas's, is making more than one new departure. He proposes to be "at home" to the medical staff and professors of the hospital on the 18th inst.

MR. DANIEL FAYERWEATHER, of New York City, recently deceased, by his will leaves 2,100,000 dollars to be divided among twenty colleges in various States, and 95,000 dollars to the hospitals of New York.

IT has been decided to enlarge the hospital at West Bromwich, at a cost of about £6,000, by the addition of two circular wards to contain twelve beds each, and new laundry and sleeping apartments for the nurses.

IN celebration of the coming of age of their eldest son, Mr. Michael Biddulph, M.P., and Lady Elizabeth Biddulph propose to build a Cottage Hospital at Ledbury, Herefordshire, to which town the Biddulph estates are adjacent.

A YOUNG medical student named Dixon lately murdered his mother. The young man's hereditary record was bad: his father and two uncles had drunk themselves to death. Dixon was therefore sent to a criminal lunatic asylum.

THE Manchester Medico-Ethical Society has been discussing the high death-rate of Manchester and Salford. Dr. Collins described it as a "terrible disgrace." It was decided to ask the Mayor to convene a meeting of medical men to consider the subject.

THE Surgical Aid Society secured the Lord Mayor to preside over its annual meeting last week. The income has been £8,534, and the number of patients assisted 8,970 in the year. It is an excellent charity, not its smallest merit being that a patient only needs to secure one "letter." Voting charities are the curse of modern philanthropy.

PROFESSOR KOCH has been elected honorary fellow of many medical societies on the Continent—e.g., at Vienna, Prague, Buda-Pesth, and elsewhere. The freedom of Wollstein in Posen, where he was in practice, has been bestowed on him, and a tablet is to be affixed to the house he occupied.

THE annual sale of work on behalf of the Tweed Bed Endowment Fund in connection with Coldstream Cottage Hospital was held within the Mechanics' Institute Buildings at Coldstream on November 29th, and, being combined with a musical and dramatic entertainment, proved in every respect a signal success.

IT is stated that the late Mr. George Angus, of Low Gosforth Hall, bequeathed by his will a considerable amount to local and other charities, including £150 to the Gateshead Children's Hospital, £150 to the Gateshead Dispensary, and £100 to the Newcastle-on-Tyne Dispensary and Newcastle Infirmary.

IT is stated that a hospital, purely for the accommodation of private patients who come to Plymouth for surgical or medical treatment (a long-felt want), is about to be provided by a number of gentlemen, chiefly members of the faculty. A site has been selected at Woodside, and the necessary steps taken for the erection of such an institution.

THE following resolution has been passed by the committee of the Royal Bath Hospital and Rawson Convalescent Home at Harrogate: "That this meeting having heard the statements of the Committee of Management with respect to the present financial position of the institution and the very great expense incurred by keeping the buildings open for the reception of patients during the winter months, hereby resolve that the hospital and home be closed from the second Thursday in November to the first Thursday in April, and that the rules be revised accordingly."

### The Student of Medicine.

[All communications for this Supplement should be addressed to The Editor of The Hospital, at 140, Strand, with the words, "Students' Column," written in the left hand top corner of the envelope.]

### EDITORIAL NOTES.

Oxford Undergraduates.—It appears from several sensational accounts that have found their way into some of the daily papers, that the students of Queen's have been indulging in what is usually termed undergraduate rowdyism. The provost of the college has refused to make any statement, or to give any account of the facts before first consulting with the other members of the college board.

A Practical Joke.—It appears that a certain freshman had not conformed to some of the unwritten laws observed by students on their entrance into college. This breach of local etiquette made him very unpopular, with the result that a practical joke was arranged to be played on him. Accordingly at about midnight of the 3rd instant, a party of undergraduates, arrayed in a variety of costumes, formed a procession and went for the rooms of their intended victim, and on arriving there found the gentleman in question asleep. Having provided themselves with various forms of musical instruments, popular on such occasions, such as baths, biscuit tins, trays, and the like, they set up a tremendous din by playing on them with sticks and other instruments, in the bedroom of the sleeping freshman. He was hustled out of bed, and probably half through fright, ran downstairs into the court, around which he was pursued, his only clothing being his nightshirt. After a few minutes exercise round the college grounds the victim returned wearied with the chase to his rooms, but minus the aforementioned covering for his skin.

The Victim Revenged.—The following morning the whole affair was reported by the gentleman deprived of his slumbers to the college authorities. An inquiry was instituted, with the result that some of the members of the college have been rusticated, and smaller punishments have been inflicted upon other culprits. The rusticated gentlemen left the University accompanied by a large number of their sympathisers, the bulk of the undergraduates considering the verdict unwarranted by the circumstances.

Competitive Diplomas.—Students of medicine all over Great Britain will be pleased to hear that the Governors of the Bristol General Hospital have repealed those rules which restricted the honorary appointments on the staff to Diplomates of the London Colleges. In future, no one will be ineligible to apply for a post on the hospital staff because he does not happen to have some diploma, given by a more or less private body in one centre of education. In all other European countries a legally qualified medical man can apply and receive an appointment on his merits, and quite irrespective of the number of letters he has a right to put after his name, or of the amount of fees he has paid to various examining bodies. One student of medicine lays out many years of his earlier career in order to get the fashionable number of diplomas. Another spends the same years much more profitably to himself and to the profession at large, in making himself acquainted with many subjects outside the narrow schedules of examinations. Although the former may have no other recommendations, he is eligible for the highest hospital and other appointments, if, after repeated failures, he has at last obtained what are considered the highest diplomas. The latter, on the other hand, who may have done a large amount of original work, is ineligible even to apply for appointments. The sooner the spirit of reform of the Bristol Hospital extends to other institutions, the better for the advancement of British Science, and for the medical profession at large.

The Pavy Gymnasium.—The Guy's students are fortunate in having Dr. Pavy as a member of the hospital staff. The new gymnasium, which was presented by the doctor, is certainly a great boon to the college men. Built in, or rather sunk into, the courtyard of the residential College, it is very conveniently situated. It has been most perfectly fitted up by its donor, and contains most of the modern apparatus for physical development. An excellent description of the gymnasium, together with a list of the apparatus and fittings, written by Mr. W. Bligh, the hon. secretary, is published in the *Guy's Hospital Gazette* for October 11th.

### APPOINTMENTS.

At the Hospital for Consumption, Brompton, Sidney Allden, M.D. Durham, B.S., L.R.C.P., M.R.C.S., has been appointed assistant resident medical officer; J. E. Kershaw, M.B. Oxon., L.R.C.P. Lon., M.R.C.S., has been appointed resident medical officer. At St. Bartholomew's Hospital, F. C. Wallis, B.A., M.B., B.S., has been appointed assistant demonstrator of anatomy. At the London Hospital, F. R. Riley, M.R.C.S., L.R.C.P., has been appointed house surgeon. At the Sick Children's Hospital, Aberdeen, R. A. S. Eden, B.B., C.M. Aberdeen, has been appointed house physician and surgeon. At the College of Medicine, University of Durham, Newcastle-on-Tyne, Philip R. W. Santi, F.R.C.S., L.R.C.P. Lon., has been appointed senior demonstrator of anatomy. At the Carnarvonshire and Anglesey Infirmary, Bangor, J. E. Thomas, M.B., C.M. Edin., has been appointed house surgeon. At Dr. Barnardo's Home for Children, Hawkhurst, Kent, Catherine Mary Wickham, L.R.C.P., L.R.C.S. Edin., has been appointed resident medical officer. At the Monktown Hospital, Ninian McIntire Falkiner, A.B., M.B., L.R.C.P.I., has been appointed resident medical officer. At the City of London Hospital for Diseases of the Chest, Victoria Park, A. Glaster, M.D. Bucharest, L.R.C.P. Lon., has been appointed house physician. At the London Hospital, S. P. Snook, M.R.C.S. Edin., L.R.C.P. Lon., has been appointed house surgeon.

### STUDENTS' MEDICAL SOCIETIES.

### Liverpool Dental Hospital Students' Society.

A meeting of this society was held on Wednesday, November 19th. Mr. R. Edwards, president, was in the chair, and a large number of members were present. The following gentlemen were elected members: Messrs. H. P. Harding, J. H. Burroughs, S. A. Westerton, D. W. Parsons, E. C. Dilcock, J. Snape, W. E. Partridge, and J. E. Edwards. Mr. Bates showed some skulls and afterwards presented them to the museum. Mr. Norris showed some models of regulation cases. The secretary then read a letter from Mr. E. J. M. Phillips presenting on behalf of Mr. James B. Lloyd a pair of anatomical specimens to the museum. A paper on Past and Present Dentistry was then read by Mr. Hitchon and afterwards discussed by Messrs. Edwards, Osborn, Mansell, Bates, Gilmour, Parsons, and Dunn. After a vote of thanks to Mr. Hitchon, the chairman announced next meeting for December 17th.

### Guy's Hospital Pupils' Physical Society.

A meeting was held on Saturday, November 29th, Mr. J. M. Gill, M.B. in the chair. Mr. F. G. Hopkins, B.Sc., proposed a motion to the effect that if the Medical curriculum were increased by another year, it should be devoted to practical study of the Experimental Sciences rather than strictly professional subjects. Mr. T. R. Taylor, B.Sc. opposed. The discussion was continued by Messrs. W. K. Steele, A. T. Rake, T. B. P. Davies, R. Henderson, D. S. Long, E. H. Cartwright, W. T. B. Donnelly, C. F. Wakefield, Guy Mackeson, and A. J. Sharp. Mr. Hopkins having briefly replied, a show of hands was taken, 23 voting for the motion and 28 against.

### Middlesex Hospital Medical Society.

A meeting was held on November 27th, Mr. A. Pearce Gould in the chair. Mr. J. W. Williams read a paper entitled "Health in its Relation to Morals." The chief object of the paper was to show that, in a great sense, morality was dependent on the influence of environment and heredity, especially as obtained under good sanitary conditions. He criticised Dr. B. W. Richardson's proposed city of Hygeia chiefly as regards the traffic accommodation and lighting of the streets, and projected another imaginary city—the city of Ethica. In this all possible sanitary and moral conditions would be fulfilled by the instruction of the people in what should be recognised as the moral code, and in the education of the children on Froebel's system, as well as in the laws of hygiene. Considerable discussion followed the reading of the paper, the project being generally considered to be impracticable. It was argued by many that physical well being, whilst a factor in the production of morality, was far from being the only factor, whilst others pointed out the impossibility of a universal moral code. Mr. Holding then showed three drawings illustrative of Dichotomy, the president briefly alluding to the chief facts which at present are known about the subject.

### NOTES FROM THE SCHOOLS.

### BERLIN.

The students of Berlin are to hold a torchlight procession on the 21st inst. in honour of Dr. Koch.

### CAMBRIDGE.

The Thruston prize, of the value of £54, has been awarded to William Ewart, M.D., assistant physician to St. George's Hospital, London.

Professor MacAlister has been appointed chairman of examiners for the Natural Science Tripos.

F. W. Burton (St. John's), house physician to Addenbrooke's Hospital, has kept the Act for the M.B. degree, the title of his thesis being, "That the Urine Affords Valuable Indications of Certain Conditions of the Alimentary Tract."

### Notes from the Schools.—(*Continued.*)

#### OXFORD.

The students of Oriel College gave a smoking concert in the Hall on Saturday, December 6th, which was a great success. The entertainment, which was of an exceedingly varied nature, was continued nearly till midnight.

The Christmas vacation commenced on December 6th, many of the colleges went down on that day, and the remainder followed on Monday and Tuesday.

#### EDINBURGH.

The Union Billiards Championship for this year has been won by Lishman, who so becomes the holder of the twenty-five guinea trophy subscribed for by the members of the Union.

The annual general meeting of the members of the Union was held on December 5th. Dr. H. G. Melville, president of the Union, was in the chair. Sir William Muir, Principal of the University, Professor T. R. Fraser (Dean of the Faculty of Medicine), and Professor Butcher were present. The hall was well filled with student-members. The secretary's report was read, and Sir William Muir, in moving the approval of it, said that it was a pity that arts students had not joined the Union in the numbers that medical students had. The treasurer's report was read, and Professor Fraser, in moving the approval of the report, said that, considering the Union was just entering into the second year of its life, it was a very satisfactory one. In the election for the president, Mr. W. Campbell Lahore (Divinity) defeated Mr. A. Stodart Walker (Medicine) by the bare majority of 15. Mr. J. G. C. Scott was unopposed for the secretaryship.

---

### PASS LISTS.

#### Society of Apothecaries of London.

The following candidates have passed the examination in surgery:—

G. M. Arkle, Liverpool University College; T. S. Byass, University College; I. George, King's College: W. Hawker, Cambridge University and Middlesex Hospital; T. C. Hughes, Westminster Hospital; B. Nauth, L.M.S., Lahore and Punjab University; H. F. Nunes, St. Mary's Hospital; G. P. Smith, Guy's; C. T. Standring, King's College; H. J. Thomas, Bristol; R. L. Thomas, University College; F. Turner, University College; J. Wells, London Hospital; F. W. Welstead, Guy's.

The following have passed in medicine, forensic medicine, and midwifery:—

W. L. W. Buss, Middlesex Hospital; F. L. Dick, Royal Free Hospital; L. F. Dods, St. Bartholomew's: E. J. Finch, St. Mary's Hospital; G. Garrard, St. Mary's Hospital; C. E. M. Hey, B.A., Cambridge University and St. Mary's Hospital; C. M. Leakey, London Hospital; A. E. Leitch, Liverpool University College; R. H. D. Mahon, St. Thomas's; J. S. Tabb, Charing Cross Hospital; R. L. Thomas, University College; H. A. Vernon, London Hospital; H. Watts, London Hospital.

The following passed in the subjects as indicated:—

Medicine and forensic medicine—F. W. Mawby, Guy's; H. F. Nunes, St. Mary's Hospital. Medicine and midwifery—F. B. Shaw, Middlesex Hospital. Medicine—H. Fairfax, Charing Cross Hospital; A. W. C. Herbert, St. Mary's Hospital. Forensic medicine—G. M. Arkle, Liverpool University College.

The following, having passed the qualifying examination in medicine, surgery, and midwifery, were granted the diploma of the society entitling them to register and to practise in the same:—

F. L. Dick, L. F. Dods, G. Garrard, C. E. M. Hey, B.A., Cambridge, C. M. Leakey, R. H. D. Mahon, R. L. Thomas, H. A. Vernon, H. Watts, J. Wells, F. W. Welstead.

#### University of Edinburgh.

The following gentlemen received the degrees of M.B. and C.M. on Nov. 29th:—

Duncan Drummond, Comrie, Perthshire; Herbert H. Hearsey, Inner Temple, London; Wm. S. Kerr, Dumfries; Henry J. Mackenzie, Mayfield Road, Edinburgh; John Welsh Smith, Dalry, Galloway; Wm. W. Williamson, Lymm, near Warrington.

#### University of London.

M.B. EXAMINATION, OCTOBER, 1890.

EXAMINATION FOR HONOURS.

MEDICINE.—*First Class*: T. L. Pennell, B.Sc. (scholarship and gold medal), University College; J. H. Bryant (gold medal), Guy's Hospital; P. C. Evans, University of Edinburgh. and Guy's Hospital; F. C. Abbott, B.Sc., St. Thomas's Hospital; Alice McLaren, London School of Medicine for Women; F. Grange, Charing Cross Hospital; H. J. Waring, B.Sc., St. Bartholomew's Hospital. *Second Class*: Annette M. Benson, B.Sc., London School of Medicine for Women; J. H. Sequeira, London Hospital; S. H. Snell, University College; A. W. W. Lea, Owens College and Manchester Royal Infirmary; R. C. Bailey, St. Bartholomew's Hospital; C. J. Martin, B.Sc., St. Thomas's Hospital; H. Tilley, University College. *Third Class*: H. G. G. Cook, St. Bartholomew's Hospital; J. S. Richards, Guy's Hospital.

OBSTETRIC MEDICINE.—*First Class*: A. W. W. Lea (scholarship and gold medal), Owens College and Manchester Royal Infirmary; F. C. Abbott (gold medal), St. Thomas's Hospital; J. S. McGowan, B.Sc., Owens College and Manchester Royal Infirmary; P. C. Evans, Univer-

**Pass Lists**—(*continued.*)

sity Edinburgh and Guy's Hospital. *Second Class:* J. S. Richards, Guy's Hospital; J. Moore, St. Bartholomew's Hospital; F. W. Hall, Guy's Hospital; W. McAdam Eccles, St. Bartholomew's Hospital; S. H. Snell, University College; E. V. Hugo, St. Bartholomew's Hospital; J. D. E. Mortimer, St. Bartholomew's and Westminster Hospitals; J. Robertson, Guy's Hospital; J. H. Sequeira, London Hospital; D. Brown, B.Sc., London Hospital. *Third Class:* L. Williams, University College; J. Fawcett, Guy's Hospital; R. H. Elliott, St. Bartholomew's Hospital; H. Tilley, University College.

Forensic Medicine.—*First Class:* A. W. W. Lea (scholarship and gold medal), Owens College and Manchester Royal Infirmary; *T. L. Pennell (gold medal), University College; †F. C. Abbott, St. Thomas's Hospital; F. Grange, Charing Cross Hospital; C. J. Martin, St. Thomas's Hospital; J. H. Bryant, Guy's Hospital; J. S. McGowan, Owen's College and Manchester Royal Infirmary. *Second Class:* W. McAdam Eccles, St. Bartholomew's Hospital; H. J. Waring, St. Bartholomew's Hospital; H. G. G. Cook, St. Bartholomew's Hospital; R. T. Hewlett, King's College. *Third Class:* R. H. Elliot, St. Bartholomew's Hospital; T. G. Stevens, Guy's Hospital; L. Williams, University College; P. C. Evans, University Edinburgh and Guy's Hospital; E. V. Hugo, St. Bartholomew's Hospital; E. N. Tribe, London School of Medicine for Women.

* Obtained the number of marks qualifying for the University scholarship.

† Obtained the number of marks qualifying for a gold medal.

### ATHLETICS.

The first annual smoking concert of the Dental Hospital Athletic Club took place in the French Room at St. James's Restaurant on Saturday, December 6th, Mr. Morton Smale in the chair. The programme was very well arranged and excellently varied, consisting of many comic songs, such as "The Soldier," sung by Mr. A. May; "Scissors," by Mr. R. Herschell; "Up to Date," by Mr. H. E. Bullen; together with "Laughing Song," and "Skating," by the same gentlemen. The Musical Society sang a glee entitled "Cruiskeen Lawn," and banjo duets were played by Messrs. O. C. Sibley (Middlesex), and R. Waller; Mr. O. C. Sibley also playing a banjo solo entitled "Home, Sweet Home," with variations. The smoker was voted a great success, and should be a stimulus for the athletic club of the Dental Hospital to give another one.

### Football.

Association Rules, December 3rd.

*King's College Hospital v. Old Rossallians.*—This match was played at Wormwood Scrubbs in a thick fog, and the ground was very slippery. From the commencement it was seen that the teams were well matched, Dixon, Dillon, and Hetherington being conspicuous for their side, whilst, amongst others, Hutton, J. B. Toone, Nelson, and Frost, played in capital form. After a short time a rush was made on the Old Rossallians' goal, which was captured, after some resistance, by Dillon. On changing ends the play became particularly fast, but the Rossallians got the best of the struggle, W. P. Toone putting the ball through after a sharp scrummage in front of the goal. At the call of "Time" each side had secured one goal, and it being too dark to play longer the game terminated in a draw. Sides—King's College Hospital: D. Castagnana (goal), E. W. Timmiss, and H. W. Lyle (backs), E. G. Storrs, A. N. Rock and C. Wace (half-backs), R. T. Tanner and E. H. Dixon (right wing), C. F. Backhouse (centre), L. C. Dillon and G. M. Hetherington (left wing). Old Rossallians: M. R. Taylor (goal), H. A. Hutton (captain) and W. Benley (backs), J. B. Toone, W. B. Bell, and J. Nelson (half-backs), W. Frost, E. Snow. W. P. Toone, N. Golland, and Miles.

*Cambridge University v. Norfolk.*—This match was played at the Corpus Ground, Cambridge, in the presence of a small company. The University had a very weak team, which only included two blues, whilst the visitors were fairly represented. It was not a very brilliant game, and the light blues were the victors by three goals to nothing. The University set the ball rolling at a quarter to three, and immediately forced the play into close proximity to the visitors' goal; soon after it was returned by the Norfolk men. The blues next caused their rivals to act on the defensive, and Ll. Davies was successful in kicking the goal, ten minutes' play having elapsed. Commencing again, a capital shot was sent in by J. F. Fernie, which was stopped by the goalkeeper. Soon afterwards W. F. H. Stanbrough succeeded in landing the ball through the uprights. This was afterwards transferred to the other end of the field, and shots were sent in by M. H. Stanborough, Ll. Davies, E. C. Streatfeild, and N. C. Cooper, but none had the desired effect. At half-time the home team crossed over with two goals to the good. Ll. Davies soon sent in a rattling shot, which hit the goal post. Just before the call of "No side" G. P. Dewhurst obtained another goal for the University, and they thus left the field victorious, as stated above. Umpires: E. F. Halstead (Trinity) and R. Borrett (Norfolk); referee: H. A. Harrison (Trinity). Sides:—Cambridge University.—S. W. Morgan (Caius) (goal), *J. R. Paull (Pembroke) and F. J. Kittermaster (King's) (backs), W. K. Fernie (Clare), N. C. Cooper (Jesus), and E. C. Streatfeild (Pembroke) (half-backs), W. F. H. Stanbrough (Trinity) and G. P. Dewhurst (Trinity) (right wing), Llewellyn Davies (Christ's) (centre), H. Pearce (Trinity) and *M. H. Stanbrough (Caius) (left wing). Norfolk.—T. Morley (goal), H. Morgan-Brown and H. C. Scotter (backs), T. C. Sneath, J. Bates, and E. Orams (half-backs), E. H. Fryer and T. H. Brown (right wing), J. F. Fernie (centre), P. Ives and N. Perkins (left wing). * Signifies Blues.

The match between the *Royal Military Academy* and *St. Thomas's Hospital*, fixed for Woolwich yesterday, was postponed.

December 6th.

*Oxford University v. Old Westminsters.*—This match was played at the Surrey Cricket Ground in the presence of a good number of visitors, as

### Athletics—*continued.*

it was the first appearance at the Oval of the Oxford eleven this season. The game was finished in a very bad light, owing to the delay before the ball was kicked off. The Old Westminsters were the first to show to advantage, but a good run down the middle enabled the University to assume the offensive, but the only result was a corner kick, which was fruitless. The Oxonians had again to retreat, and this time the Westminster attack was successful, Hurst kicking the first goal. Shortly after Veith gained them another point. Before half-time the Oxford goal had once more fallen, the Westminsters being to the fore, led by Probyn, Sandilands, and Veitch. After changing ends the University played up better, and the game was more even. The Oxford forwards were the first to become dangerous, and Wilson succeeded in getting the ball past Moon. The combination of the Oxford eleven in the second half showed a great improvement, and they played up with great determination. The Westminsters were victorious by three goals to one. Mr. C. W. Grant Wilson was umpire for both sides. The teams were: Old Westminsters.—W. R. Moon (goal), R. T. Squire and C. J. M. Fox (backs), W. M. Winckworth, H. Wetton, and J. P. Paul (half-backs), P. C. Probyn, R. R. Sandilands, A. R. Hurst, J. G. Veitch, and Rev. E. H. Alington. Oxford University.—M. C. Elliott (goal), A. W. Malden and F. Cobbett (backs), H. C. Izard, N. F. Shaw, and R. Montgomery (half-backs), G. L. Wilson, F. Street, H. A. Rhodes, G. R. Wood, and G. W. Reeve.

The following matches were also played on December 6th: *St. Bartholomew's Hospital* beat *London Welsh*, at Dulwich (five goals to one); and *Bartholomew's v. West End*, at Shepherd's Bush, drawn (two goals to two. *Westminster Hospital* beat *Eltham House*, at Raynes Park (one try, four minors to nil).

Rugby Rules, December 1st.

*Cambridge University* were defeated by the *Hartlepool Rovers*, at Cambridge, by two goals and a try to one goal and a try.

December 4th.

*Newport* beat *St. Thomas's Hospital* by six tries to a goal, at Newport.

December 6th.

*St. Thomas's Hospital* were beaten by *Swansea*, at Swansea, by one goal and four tries to nil. *London Hospital* beat *Sidcup*, by one goal and two tries to nil. *St. Mary's Hospital* were defeated by *Windsor*, at Windsor, by one goal and three tries to nil. *St. George's Hospital* beat *the Crusaders*, at Ealing, by one goal and four tries to nil. *Middlesex Hospital* were beaten by *Brighton*, at Brighton, by three tries to nil. *Guy's Hospital* visited Northampton, and were defeated by the home team by one try to nil. *University College Hospital v. Wimbledon*, drawn (one minor to nil). *University College Hospital v. Battersea*, drawn. *Dental Hospital* were beaten by *Wandsworth*, at Kilburn (one try to nil).

### United Hospitals Hare and Hounds.

The members' short distance, which was postponed from November 29 on account of the snow, was held on Saturday, December 6, over the short distance course. Only twelve members faced the starter. Trail was laid by F. E. Easton and E. L. Payne (Mary's). The course, which is a good five miles in length, was pretty easy, and a good time was accredited to the winner. From the start the scratch men forced the running, and soon overhauled their men. Cooper and Wilde led nearly all the way, with Munro, the scratch man, close behind. Eventually victory rested with A. N. Wilde, who beat Cooper by about a minute, Cooper being well ahead of Munro, who was in turn just in front of G. E. Mould. The result was: 1, A. N. Wilde, Bart.'s (30 sec. start), time, 33 min. 25 sec.; 2, J. W. A. Cooper, King's (1 min.), 34 min. 52 sec.; 3, H. A. Munro, Guy's (scratch), 34 min. 45 sec.; 4, G. E. Mould, Mary's (1 min. 45 sec.), 37 min.; 5, W. H. Orr, Bart.'s (1 min. 45 sec.), 40 min. 15 sec. The following also ran: B. C. Green, W. B. Mercer (Bart.'s), F. G. Mould, C. H. Mathews, Lambert (Mary's).

Edinburgh.

The University Hare and Hounds Club held a club run from Musselburgh on December 6th.

The University Rugby football team start on their English tour on December 9th. Their head-quarters are to be in Manchester.

The Volunteer Medical Staff Corps had a march out on November 29th. The annual dinner of the corps was held on December 5th.

---

At Cambridge, the University Hare and Hounds contested a cross-country match with a team of the Blackheath Harriers. The result was a victory for Blackheath, although T. Colbatch-Clark, of Trinity College, secured the first individual place for the University.

### London Volunteer Medical Staff Corps.

Four companies of this corps mustered last week in the Guildhall to compete for the challenge shield of the corps, which is held for a year by the company coming out first in a drill and bandaging competition. Surgeon Dorman, of the Army Medical Department, was the judge. At the close of the contest Surgeon Dorman announced that the best results had been attained by No. 4 Company, under Surgeon-Major Watson, and composed chiefly of laymen drawn from the Birkbeck and Polytechnic Institutes.

### EVENTS FOR THE MONTH OF DECEMBER.

[Items intended for this column must reach the office, 140, Strand, W.C., not later than first post on Monday mornings.]

#### December Meetings and Athletics.

For list of Students' Medical Societies' Meetings and Football Matches during December, see The Hospital for Nov. 29th.

### General Charities.

[This Column is devoted to an account of work of charitable institutions other than Medical Charities. The Editor will be glad to receive reports or news of work at 140, Strand. *Philanthropy* should be plainly written on the envelope.]

At the general court of **THE CLERGY ORPHAN CORPORATION** held last week, twelve boys and five girls were elected to the orphan schools of the corporation. There were sixteen unsuccessful candidates, seven boys and nine girls. The boys' school is at St. Thomas' Hill, Canterbury, and the girls' at St. John's Wood. The children at these schools receive their education, clothing, and maintenance gratuitously. On leaving school they are assisted to make a start in life. Nearly three thousand fatherless children of the clergy have already been admitted to the schools, the number in which has been raised to two hundred. Secretary, Rev. H. Wesley Dennis, 62, Lincoln's Inn Fields, W.C.

The report presented at the annual meeting of the **WORKING LADS' INSTITUTE, WHITECHAPEL,** which was held last week under the presidency of the Lord Mayor, shows a falling off of subscriptions during the past year. The total number of members who have joined the institute since it was started is 5,122, and the number of members during the past twelve months was 300. The institute forms a club, college, playground, and home in one. Beneath the hall is a large room which in winter is used as a gymnasium, and in the summer as a swimming bath. The physical training and exercise afforded in such an institute must undoubtedly have a beneficial effect upon dwellers in the East End. Hon. sec., Mr. Henry Hill.

Lieut.-Colonel H. W. Appleby, commandant of the discharge depôt at Gosport, appeals for aid in his endeavour to obtain employment during the next six months for **DISCHARGED SOLDIERS** of good character. During the trooping season, which lasts from November 1st to the close of May, about 8,000 men will pass through the depôt and be sent to their homes. Colonel Appleby will register the names of any employers of labour who may apply to him for servants and workmen, and, on being notified as to the class of man required and the rate of wages offered, will select the best men, and submit their names for approval. This work will in no way be carried on in opposition to the National Association for the Employment of Reserved and Discharged Soldiers, but rather as supplementary to and in conjunction with it.

A new centre of philanthropic effort has been formed in Lancashire. A **RESCUE HOME FOR WOMEN** has recently been opened in Thurlow Street, Eccles New Road, Salford. The house, which was formerly a private residence, is well adapted for its purpose and has accommodation for fifty inmates. At present twenty beds are occupied. Mrs. A. D. Weigall is the honorary superintendent.

The managers of **THE SCHOOL FOR THE INDIGENT BLIND,** St. George's Fields, Southwark, appeal for funds for the junior branch school at Wandsworth Common. Candidates for the benefits of this charity must not be less than seven or more than twenty years of age. They are maintained free of cost for about six years, during which period they are taught a trade, and to read, write, and cypher, the trade chosen being that for which the most aptitude is shown. Upwards of two hundred and twenty blind children are in the schools. Secretary, the Rev. R. P. Stickland.

It is probably not generally known that there are upwards of thirty thousand blind people in this country, and even when known it is hard to realize how much unhappiness this means. The great majority of these are poor and dependent on the charity of friends or institutions. Owing to the want of funds **THE LONDON SOCIETY FOR TEACHING THE BLIND TO READ,** to whose work we drew attention some months ago, and which claims for itself that it is the only society in the metropolitan area which extends its benefits to children who are not entirely blind, has recently had to keep vacant several beds and has been unable to provide training for as many pupils as there is accommodation. Considerable outlay in providing for the health and safety of the inmates has been incurred in building sheds for gymnastic exercise, in various sanitary improvements, and in the erection of external staircases as a means of escape in case of fire, which has necessitated an inroad upon their invested funds. This is a form of expenditure to which few will be inclined to object. The institution gives instruction to blind people of both sexes, of all ages, and of every denomination, from all parts of the country, in the elements of education, basket and sash-line making, chair caning, knitting, &c. The mats and brushes are sold at the school, to which chairs which require re-caning can be sent, and a list of goods made and sold can be had from the matron. Secretary, Captain George G. Webber, R.N.

